# Factors Associated With Physiological Dysregulation Among US Adults Aged 45 Years and Older: A Nationally Representative Study

**DOI:** 10.1155/jare/3289222

**Published:** 2026-07-24

**Authors:** Vishal Vennu

**Affiliations:** ^1^ Department of Rehabilitation Sciences, College of Applied Medical Sciences, King Saud University, Riyadh, Saudi Arabia, ksu.edu.sa

**Keywords:** aging, biomarkers, NHANES, obesity, physical activity, physiological dysregulation

## Abstract

**Objectives:**

To examine associations of demographic, behavioral, and clinical factors with physiological dysregulation across cardiometabolic, renal, and hepatic functions among United States (US) adults aged 45 years and older using nationally representative data.

**Methods:**

This cross‐sectional study analyzed data from the National Health and Nutrition Examination Survey (NHANES) 2017–March 2020 prepandemic cycle (weighted *N* ≈ 17,219,945; analytic *n* = 496). Eight biomarkers representing cardiometabolic (glucose, total cholesterol, and triglycerides), renal (serum creatinine and blood urea nitrogen), and hepatic (alanine aminotransferase, aspartate aminotransferase, and total bilirubin) functions were standardized using survey‐weighted *z*‐scores. They averaged with equal weighting to construct a physiological dysregulation index (PDI). Higher PDI scores indicate greater physiological dysregulation. Survey‐weighted multivariable linear regression models were used to examine associations between PDI and sociodemographic, behavioral, and clinical factors.

**Results:**

Female sex was associated with significantly lower physiological dysregulation compared with male sex (*β* = −0.302, SE = 0.066, *p* < 0.001). Physically active individuals also had lower dysregulation scores than inactive individuals (*β* = −0.186, SE = 0.069, *p* = 0.007). Overweight (*β* = 0.108, SE = 0.043, *p* = 0.012) and obesity (*β* = 0.191, SE = 0.087, *p* = 0.028) were associated with greater dysregulation than normal body mass index (BMI).

**Conclusions:**

Female sex and physical activity were associated with lower physiological dysregulation, whereas higher BMI was associated with greater dysregulation. These findings underscore the importance of sex differences and modifiable lifestyle factors, particularly physical activity and adiposity in multisystem physiological burden among older US adults.

## 1. Introduction

Aging is accompanied by progressive physiological changes across multiple organ systems that contribute to increased vulnerability to chronic disease, functional decline, and mortality [1]. Although chronological age remains a useful population‐level indicator, substantial heterogeneity exists in physiological health among individuals of the same age [2–4]. Consequently, there is increasing interest in identifying biomarker‐based approaches that capture variations in physiological status beyond chronological age [5–8].

Composite biomarker indices provide a practical approach for summarizing physiological burden across multiple organ systems using routinely collected clinical measures. Such indices have been used to characterize multisystem dysregulation and examine associations with adverse health outcomes in population‐based studies. Their utility is particularly relevant for public health surveillance because they can be derived from laboratory measures that are widely available and comparable across settings [9–12].

In the United States (US), the growing burden of obesity, diabetes, cardiovascular disease, and other noncommunicable diseases highlights the need for approaches that can identify individuals experiencing elevated physiological burden [10, 13]. Previous studies [14–18] have suggested associations between adiposity, physical inactivity, smoking, and physiological dysregulation. However, evidence on demographic and lifestyle correlates of physiological dysregulation remains inconsistent, particularly in nationally representative US populations. Therefore, this study aimed to examine the associations of demographic, behavioral, and clinical factors with physiological dysregulation across cardiometabolic, renal, and hepatic function among US adults aged 45 years and older using nationally representative data.

## 2. Materials and Methods

### 2.1. Study Design and Data Source

This cross‐sectional study used publicly available data from the National Health and Nutrition Examination Survey (NHANES) 2017–March 2020 pre‐pandemic cycle, conducted by the National Center for Health Statistics (NCHS). NHANES is a nationally representative survey of the noninstitutionalized US population employing a complex, multistage probability sampling design with oversampling of key demographic subgroups [19]. All analyses incorporated NHANES survey weights, strata, and primary sampling units (PSUs) to account for the complex sampling design and to generate nationally representative estimates. The study followed the Strengthening the Reporting of Observational Studies in Epidemiology (STROBE) guidelines [20]. NHANES data are publicly available and de‐identified; therefore, ethical approval and informed consent were not required for this secondary data analysis.

### 2.2. Study Participants

The analytic sample included adults aged 45 years and older with complete data on selected biomarkers and covariates. Participants with missing values in any of the eight biomarkers or key covariates were excluded. This restriction resulted in a final analytic sample of 496 participants. Although this represents a substantial reduction from the original eligible NHANES sample, NHANES survey weights appropriate for the combined 2017–March 2020 cycle were applied in all analyses to account for the complex sampling design, differential nonresponse, and oversampling of key demographic groups. Weighted estimates, therefore, reflect population‐level inferences among US adults aged ≥ 45 years with complete biomarker and covariate data.

### 2.3. Physiological Dysregulation Index (PDI)

The primary outcome of interest was a PDI, constructed using eight routinely available biomarkers representing cardiometabolic (glucose, total cholesterol, and triglycerides), renal (serum creatinine and blood urea nitrogen), and hepatic (alanine aminotransferase [ALT], aspartate aminotransferase [AST], and total bilirubin) function. These biomarkers are routinely measured in NHANES laboratory components and have been collected across multiple survey cycles. This enables potential comparability of biomarker distributions across survey waves at the population level. However, because NHANES is designed as a repeated cross‐sectional survey rather than a longitudinal cohort, temporal comparisons reflect population‐level trends rather than within‐individual changes over time.

Each biomarker was first oriented so that higher values reflected poorer physiological status. For biomarkers in which lower values are generally indicative of poorer physiological status (e.g., total bilirubin), values were reverse‐coded prior to standardization so that higher standardized scores consistently reflected greater physiological dysregulation across all biomarkers. To ensure interpretability, survey‐weighted means and standard deviations (SDs) were then used to standardize each biomarker into *z*‐scores before aggregation. Specifically, each value was transformed as (*x* − *μ*_weighted)/*σ*_weighted, where *μ*_weighted and *σ*_weighted were estimated using NHANES survey weights for each biomarker separately. Standardized biomarker values were then combined using an equal‐weighted arithmetic mean across all eight biomarkers to generate the PDI: $PDI_{i}=\left(18/\right)\sum_{j=1}^{8}Z_{ij},$ where *Z*_*i* *j*_ represents the standardized value of biomarker *j* for participant *i*. An equal‐weighted approach was used to avoid imposing differential weighting assumptions across biomarkers and to ensure reproducibility in population‐based settings. The PDI was analyzed as a continuous variable, with higher values indicating greater physiological dysregulation [10–12, 21, 22]. While this approach enhances transparency and reproducibility, it assumes equal contribution of each physiological domain and does not account for potential differences in relative biological importance.

### 2.4. Factors

The exposure factors were selected a priori based on established associations with cardiometabolic health and physiological function. Demographic variables included age (continuous and categorized: 45–54, 55–64, 65–74, ≥ 75 years), sex (male, female), race/ethnicity (Mexican American, Other Hispanic, Non‐Hispanic White, Non‐Hispanic Black, Non‐Hispanic Asian, and Other/Multiracial), and education (< 9th grade, 9–11th grade, high school, college, and graduate). Behavioral variables included smoking status (current smoker, non‐smoker), alcohol use (yes or no), and physical activity (active, inactive). Clinical variables included body mass index (BMI), categorized as underweight (< 18.5 kg/m^2^), normal weight (18.5–24.9 kg/m^2^), overweight (25.0–29.9 kg/m^2^), and obesity (≥ 30 kg/m^2^). Clinical variables also included self‐reported hypertension (yes/no) and diabetes status (yes/no).

Physical activity was assessed using the Global Physical Activity Questionnaire (GPAQ), which recorded weekly engagement in vigorous and moderate work, recreational activities, and transportation methods like walking or bicycling [23]. Vigorous activities comprised tasks such as heavy lifting, construction, jogging, or basketball that significantly raised breathing or heart rate for at least 10 min nonstop. Moderate activities included brisk walking, light load carrying, swimming, or volleyball, causing a modest increase in breathing or heart rate for at least 10 min. Walking and cycling were specifically assessed as transportation activities performed continuously for 10 min or more. Weekly physical activity energy expenditure was calculated by multiplying the duration and frequency of moderate‐ and vigorous‐intensity activities by their respective metabolic equivalent (MET) values (4 METs for moderate activities and walking/cycling; 8 METs for vigorous activities). Total weekly MET‐minutes were calculated by summing MET‐minutes across all activity domains. Participants were subsequently categorized according to the weighted distribution of weekly MET‐minutes. Individuals with weekly MET‐minutes at or above the 75th percentile (upper quartile) were classified as physically active, whereas those below this threshold were classified as inactive. This data‐driven categorization was selected to distinguish participants with relatively high physical activity levels within the study population while maintaining reproducibility.

### 2.5. Statistical Analysis

Statistical analyses were conducted in R, version 4.3.3 (R Foundation for Statistical Computing, Vienna, Austria). Survey analyses were performed using the survey package (version 4.4), data management was conducted using dplyr (version 1.1.4) and tidyr (version 1.3.1), and figures were generated using ggplot2 (version 3.5.1). Survey weights, strata, and PSUs were applied in all analyses using appropriate survey‐weighted procedures. Weighted descriptive statistics were computed for all variables. Continuous variables were summarized as weighted means and SDs, and categorical variables as weighted proportions. Kernel density plots were used to visualize the distribution of PDI across the study population. PDI distributions were also summarized by age group and sex. These estimates represent nationally representative distributions among US adults aged ≥ 45 years with complete biomarker data.

Associations between PDI and demographic, behavioral, and clinical variables were examined using survey‐weighted multivariable linear regression models. PDI was modeled as a continuous dependent variable. The multivariable models included age, sex, race/ethnicity, education, smoking status, alcohol use, physical activity, BMI category, hypertension, and diabetes status. This model incorporated NHANES survey weights to ensure design‐consistent estimates under correct model specification. Regression coefficients (β) and standard errors (SEs) were reported. Statistical significance was defined as a two‐sided *p*‐value < 0.05.

## 3. Results

### 3.1. Participant Selection

**FIGURE 1:**
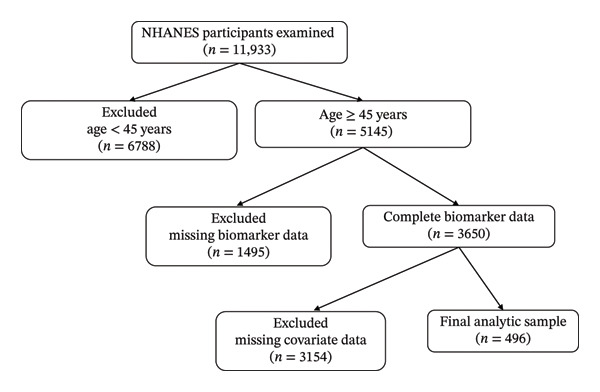
Flow diagram of participant selection for the analytic sample. Note: exclusion criteria included missing biomarker measurements and incomplete covariate information. The final analytic sample consisted of adults aged 45 years and older with complete data on all biomarkers and covariates used in the analysis.

Figure 1 presents the participant selection process. Of 11,933 total participants identified in NHANES 2017–March 2020, 6788 were excluded because of being < 45 years of age, 1495 because of missing biomarker measurements, and 3,154 because of incomplete covariate information, yielding a final analytic sample of 496 participants representing approximately 17 million US adults. This flow diagram presents unweighted sample sizes reflecting participant inclusion and exclusion, whereas all descriptive statistics and regression analyses incorporate NHANES survey weights to generate nationally representative estimates.

### 3.2. Population Characteristics

The study included a weighted population of 17,219,945 US adults aged ≥ 45 years, with a mean age of 60.4 years (SD: 9.3). Men comprised 58.6% of the population and women 41.4%. The majority were Non‐Hispanic White (69.8%), followed by Non‐Hispanic Black (8.6%), Other Hispanic (8.3%), Other/Multiracial (7.6%), Mexican American (2.9%), and Non‐Hispanic Asian (2.8%). Educational attainment was generally high, with 65.7% having attended college or graduated from college. Most participants were nonsmokers (70.1%) and reported alcohol use (96.8%). Slightly more than half were physically active (55.2%), while 44.8% were classified as inactive. Regarding BMI, 38.1% were overweight, and 35.5% were obese, whereas 25.2% had a normal weight, and 1.2% were underweight. Hypertension was present in 40.5% of participants, and 12.5% had diabetes (Table 1).

**TABLE 1:** Survey‐weighted demographic, socioeconomic, lifestyle, and health characteristics of NHANES participants aged ≥ 45 years.

Characteristic	Weighted *N* (%) or mean (SD)
Weighted US population estimate	17,219,945
Analytical sample (unweighted)	496
Age (years)	60.41 (9.3)
Sex	
Male	10,093,821 (58.6%)
Female	7,126,124 (41.4%)
Race/ethnicity	
Mexican American	507,963 (2.9%)
Other Hispanic	1,433,511 (8.3%)
Non‐Hispanic White	12,019,001 (69.8%)
Non‐Hispanic Black	1,478,619 (8.6%)
Non‐Hispanic Asian	476,616 (2.8%)
Other/multiracial	1,304,234 (7.6%)
Education	
< 9th grade	433,449 (2.5%)
9–11th grade	849,310 (4.9%)
High school	4,627,476 (26.9%)
College	6,524,430 (37.9%)
Graduate	4,785,281 (27.8%)
Smoking	
Current	5,140,351 (29.9%)
Nonsmoker	12,079,594 (70.1%)
Alcohol use	
Yes	16,670,655 (96.8%)
No	549,290 (3.2%)
Physical activity	
Active	9,504,952 (55.2%)
Inactive	7,714,993 (44.8%)
BMI category	
Underweight	202,196 (1.2%)
Normal	4,339,980 (25.2%)
Overweight	6,559,073 (38.1%)
Obese	6,118,695 (35.5%)
Hypertension	
No	10,244,330 (59.5%)
Yes	6,975,615 (40.5%)
Diabetes	
No	15,069,477 (87.5%)
Yes	2,150,468 (12.5%)

Abbreviations: BMI, body mass index; SD, standard deviation; US, United States.

Among adults aged ≥ 45 years included in the analytic sample, weighted mean glucose concentration was 108.0 mg/dL (SD: 43.0), while mean total cholesterol and triglyceride levels were 193.0 mg/dL (SD: 45.5) and 142.0 mg/dL (SD: 101.0), respectively. Renal biomarkers demonstrated weighted mean values of 0.92 mg/dL (SD: 0.43) for creatinine and 16.1 mg/dL (SD: 5.89) for blood urea nitrogen. Liver function biomarkers showed mean ALT and AST concentrations of 22.3 U/L (SD: 15.2) and 22.8 U/L (SD: 10.2), respectively, while mean total bilirubin was 0.50 mg/dL (SD: 0.30). Several biomarkers exhibited substantial variability and right‐skewed distributions, particularly glucose and triglycerides, as reflected by their wide observed ranges (Table 2).

**TABLE 2:** Distribution of physiological biomarkers among NHANES participants aged ≥ 45 years.

Biomarker	Mean	SD	Min	Max
Glucose (mg/dL)	108.0	43.0	40	898
Total cholesterol (mg/dL)	193.0	45.5	63	454
Triglycerides (mg/dL)	142.0	101.0	24	1724
Creatinine (mg/dL)	0.92	0.43	0.38	15.2
BUN (mg/dL)	16.1	5.89	4	74
ALT (U/L)	22.3	15.2	3	211
AST (U/L)	22.8	10.2	7	155
Total bilirubin (mg/dL)	0.50	0.30	0.11	6.6

*Note:* These biomarkers were standardized as survey‐weighted *z*‐scores and combined with equal weighting to derive the physiological dysregulation index (PDI). Equal weighting was chosen because no established evidence supports differential weighting of the selected biomarkers and because equal‐weighted indices have been widely used in physiological dysregulation research.

Abbreviations: ALT, alanine aminotransferase; AST, aspartate aminotransferase BUN, blood urea nitrogen; NHANES, National Health and Nutrition Examination Survey; SD, standard deviation.

### 3.3. Distribution of Physiological Dysregulation

**FIGURE 2:**
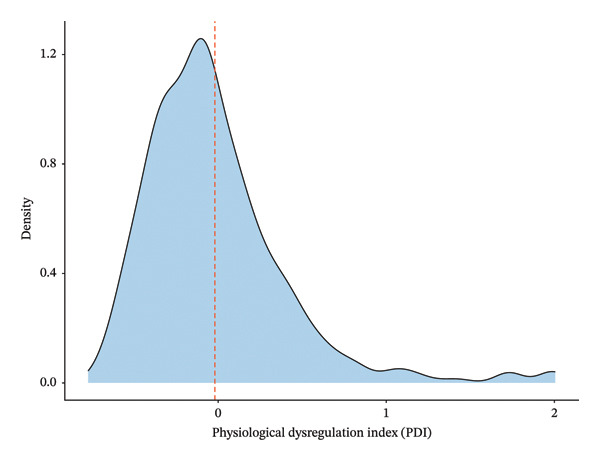
Distribution of physiological dysregulation index (PDI) among US adults aged 45 years and older. Note: The figure displays the survey‐weighted kernel density distribution of PDI scores. The dashed vertical line indicates the standardized mean (0). Higher scores indicate greater physiological dysregulation across cardiometabolic, renal, and hepatic systems.

**FIGURE 3:**
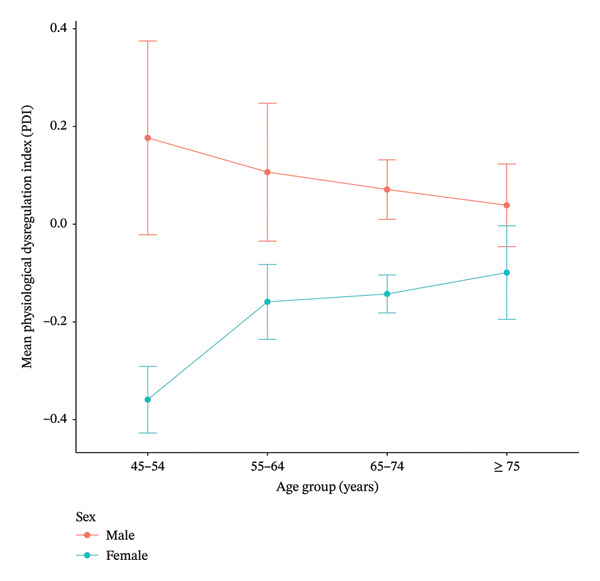
Survey‐weighted mean physiological dysregulation index (PDI) scores according to age group and sex among US adults aged 45 years and older. Note: Points represent survey‐weighted means, and error bars indicate 95% confidence intervals. Higher values indicate greater physiological dysregulation.

The distribution of PDI scores was right skewed, with most participants clustered near the standardized population mean and a smaller proportion exhibiting substantially elevated scores, indicating greater physiological dysregulation (Figure 2). In the 45–54 years age group, females had a lower mean PDI than males (−0.4 vs 0.2), a pattern that persisted across older age groups. For ages 55–64, 65–74, and 75–84, mean PDI values were around −0.2 vs 0.1 for female vs male, respectively (Figure 3).

### 3.4. Association Between Sociodemographic, Behavioral, and Physiological Dysregulation

**FIGURE 4:**
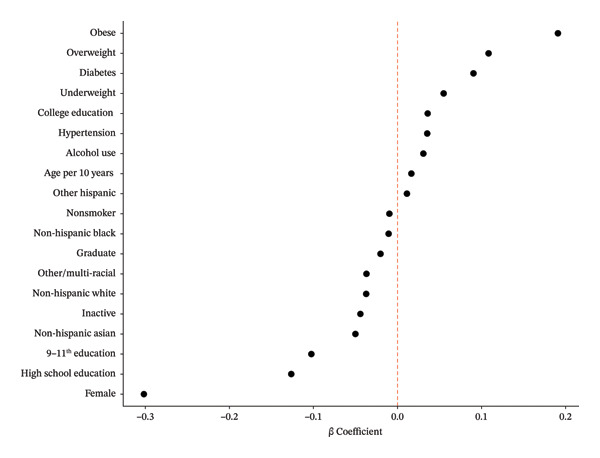
Forest plot of factors associated with physiological dysregulation Index (PDI) scores among NHANES participants aged ≥ 45 years. Note: Black circles represent the *β* coefficient.

Female participants had significantly lower physiological dysregulation scores than males (*β* = −0.302, SE = 0.066, *p* < 0.001). Physically active participants exhibited lower dysregulation scores compared with inactive individuals (*β* = −0.186, SE = 0.069, *p* = 0.007). Higher BMI was associated with greater physiological dysregulation, with overweight (*β* = 0.108, SE = 0.043, *p* = 0.012) and obese participants (*β* = 0.191, SE = 0.087, *p* = 0.028) demonstrating significantly higher scores than those with normal BMI. Age, race/ethnicity, educational attainment, smoking status, alcohol use, hypertension, and diabetes were not significantly associated with physiological dysregulation (all *p* > 0.05). Overall, sex, physical activity, and adiposity emerged as the primary independent correlates of physiological dysregulation in this population (Table 3 and Figure 4).

**TABLE 3:** Survey‐weighted multivariable linear regression analysis of factors associated with physiological dysregulation index (PDI) scores among NHANES participants aged ≥ 45 years.

Predictor	β	SE	*t*‐value	*p*‐value
Age (per 10 years)	0.017	0.025	0.66	0.507
Female vs. male	−0.302	0.066	−4.57	< 0.001
Other Hispanic vs Mexican American	0.011	0.135	0.08	0.934
Non‐Hispanic White vs. Mexican American	−0.037	0.095	−0.39	0.696
Non‐Hispanic Black vs. Mexican American	−0.011	0.122	−0.09	0.931
Non‐Hispanic Asian vs. Mexican American	−0.050	0.183	−0.27	0.785
Other/Multiracial vs. Mexican American	−0.037	0.135	−0.27	0.785
9–11th grade vs. < 9th grade	−0.103	0.082	−1.26	0.207
High school vs. < 9th grade	−0.126	0.084	−1.51	0.132
College vs. < 9th grade	0.036	0.102	0.35	0.723
Graduate vs. < 9th grade	−0.020	0.080	−0.25	0.802
Nonsmoker vs. current smoker	−0.010	0.081	−0.12	0.905
Alcohol use (Yes vs. No)	0.031	0.069	0.45	0.656
Physical activity (active vs. inactive)	−0.186	0.069	−2.71	0.007
Underweight vs. normal BMI	0.055	0.096	0.57	0.566
Overweight vs. normal BMI	0.108	0.043	2.53	0.012
Obese vs. normal BMI	0.191	0.087	2.21	0.028
Hypertension (Yes vs. No)	0.035	0.037	0.95	0.341
Diabetes (Yes vs. No)	0.090	0.065	1.40	0.163

*Note:* *β* coefficient. Estimates derived from survey‐weighted linear regression models accounting for complex sampling design (weights, strata, and PSUs).

Abbreviations: BMI, body mass index; NHANES, National Health and Nutrition Examination Survey; PDI, physiological dysregulation index; PSU, primary sampling unit; SE, standard error.

## 4. Discussion

This nationally representative study examined factors associated with physiological dysregulation among US adults aged 45 years and older. Female sex and physical activity were associated with lower physiological dysregulation, whereas higher BMI was associated with greater dysregulation.

Female sex emerged as the strongest independent predictor of lower physiological dysregulation. This finding likely reflects multiple complementary mechanisms. Biologically, women generally demonstrate more favorable lipid metabolism, greater insulin sensitivity before menopause, and lower visceral adiposity than men, all of which may contribute to healthier cardiometabolic profiles [24]. Behavioral factors, including greater utilization of preventive healthcare services and healthier lifestyle practices, may further reduce multisystem physiological burden. Clinically, sex differences in body fat distribution, inflammatory pathways, oxidative stress, and hepatic metabolism may also influence the biomarker profile incorporated into the PDI [25]. Collectively, these mechanisms may explain why female participants exhibited consistently lower physiological dysregulation across age groups despite adjustment for multiple demographic, behavioral, and clinical covariates.

Physiological dysregulation across multiple systems has been linked to functional decline and mortality beyond chronological age [5, 11, 12]. Previous work has demonstrated relationships between multisystem biomarker composites and adverse outcomes in diverse populations, but direct cross‐national comparisons are uncommon. A recent comparative study of aging patterns across countries reported greater multisystem risk in low‐ and middle‐income countries relative to high‐income settings, but sample limitations hindered representativeness [26, 27]. The present findings extend this literature by identifying consistent associations between sex, adiposity, and physical activity and a composite biomarker‐based index of multisystem physiological burden in a US population.

The graded relationship observed between BMI and PDI suggests that excess adiposity may contribute to physiological burden across multiple organ systems. Obesity has been linked to chronic inflammation, insulin resistance, oxidative stress, and metabolic dysfunction, all of which may adversely affect cardiometabolic, renal, and hepatic functions [12, 28–31]. Conversely, physical activity may help preserve physiological function through favorable effects on metabolic regulation, cardiovascular health, and body composition.

Although the observed associations are largely interpreted through cardiometabolic mechanisms, the PDI also incorporates biomarkers reflecting hepatic and renal physiology. Hepatic biomarkers (ALT, AST, and bilirubin) provide information regarding hepatocellular integrity, metabolic liver function, and oxidative stress, all of which are influenced by obesity, insulin resistance, and physical activity [32, 33]. Likewise, renal biomarkers (serum creatinine and blood urea nitrogen) reflect kidney function and protein metabolism and may capture early physiological alterations associated with metabolic dysfunction and aging [34, 35]. Therefore, the observed associations likely reflect integrated physiological changes across interconnected cardiometabolic, hepatic, and renal systems rather than isolated dysfunction within any single organ.

Interestingly, diabetes status was not independently associated with physiological dysregulation after multivariable adjustment despite glucose being included in the composite index. This finding may reflect limited statistical power due to the relatively small analytic sample, heterogeneity in disease severity and treatment status among participants with diabetes, or the multidimensional nature of the PDI, which incorporates biomarkers from cardiometabolic, renal, and hepatic domains rather than glucose alone. Future studies with larger samples should further investigate the relationship between diabetes and multisystem physiological dysregulation.

Hypertension was also not independently associated with physiological dysregulation after multivariable adjustment. Although hypertension is a well‐established risk factor for cardiovascular and renal disease [36], its independent association with the PDI may have been attenuated by effective antihypertensive treatment, variation in disease duration and severity, residual confounding, and overlap with obesity and other cardiometabolic risk factors included in the regression model. Furthermore, because the PDI summarizes multiple physiological systems rather than vascular function alone, hypertension may contribute only modestly to the overall composite score when considered alongside renal and hepatic biomarkers.

Contrary to expectations, age was not independently associated with the index after multivariable adjustment in this cross‐sectional sample. This finding suggests that the index may capture current physiological burden rather than cumulative aging processes. Consequently, the PDI should be interpreted as a measure of physiological dysregulation in three domains (cardiometabolic, renal, and hepatic) rather than as a biological age estimator. Unlike validated biological aging measures such as epigenetic clocks, Phenotypic Age, or Pace of Aging metrics, the PDI is intended to summarize physiological burden across cardiometabolic, renal, and hepatic systems using routinely available clinical biomarkers.

### 4.1. Limitations

Several limitations should be considered when interpreting these findings. First, the analytic sample was restricted to participants with complete information on all biomarkers and covariates required for constructing the PDI, resulting in a final sample of 496 participants. This complete‐case approach may have introduced selection bias if excluded participants differed systematically from those included with respect to health status, socioeconomic characteristics, or behavioral risk factors. Consequently, the findings should be interpreted as representative of NHANES participants with complete biomarker and covariate information rather than the entire eligible NHANES population.

Second, because NHANES biomarker data are not missing completely at random, the exclusion of participants with incomplete biomarker profiles may limit generalizability to the full NHANES target population. However, all analyses incorporated NHANES survey weights, which partially adjust for unequal probabilities of selection, oversampling, and nonresponse, thereby preserving population‐level inference among U.S. adults aged ≥ 45 years with complete biomarker data. In addition, no sensitivity analyses (e.g., alternative biomarker weighting schemes or exclusion‐based robustness checks) were conducted, and future studies should evaluate the robustness of the composite index using alternative construction methods such as principal component‐based weighting or domain‐specific weighting approaches.

Third, the cross‐sectional design precludes causal inference and does not allow assessment of temporal relationships between physiological dysregulation and associated demographic or behavioral factors. Fourth, the PDI was constructed using biomarkers from cardiometabolic, renal, and hepatic domains only and did not include inflammatory, immune, or neuroendocrine markers that are also relevant to systemic physiological regulation. Therefore, the index reflects multisystem physiological dysregulation across cardiometabolic, renal, and hepatic domains and should not be interpreted as a direct measure of biological aging or lifespan‐related aging processes.

Fifth, important factors that may influence physiological dysregulation, including diet quality, psychosocial stress, sleep characteristics, medication use, and inflammatory biomarkers, were not included because they were beyond the scope of the present analysis. Sixth, biomarker measurements were obtained at a single time point and may be subject to short‐term biological variability, limiting the ability to capture longitudinal physiological trajectories. Seventh, behavioral covariates such as physical activity and smoking status were self‐reported and may be subject to recall and reporting bias.

Eighth, the PDI was constructed using an equal‐weighting approach, which assumes equal contribution of each biomarker to overall physiological dysregulation. While this approach enhances transparency, reproducibility, and interpretability, alternative weighting schemes may yield different estimates and should be explored in future research. Ninth, model diagnostics, including formal assessment of multicollinearity, residual normality, and heteroscedasticity, were not conducted. However, all multivariable models were estimated using survey‐weighted linear regression procedures consistent with NHANES analytic guidelines, which provide robust design‐based variance estimation. Finally, Sensitivity analyses using alternative biomarker weighting schemes (e.g., principal component‐based weighting or unequal domain‐specific weights) were not performed. Therefore, the robustness of the PDI across alternative construction methods could not be evaluated and should be examined in future studies. Despite these limitations, the strengths of this study include the use of nationally representative NHANES data, survey‐weighted analyses, and a biomarker‐based approach incorporating multiple physiological systems.

## 5. Conclusion

In this nationally representative sample of US adults aged ≥ 45 years, physiological dysregulation varied significantly by sex and modifiable lifestyle factors. Female sex and physical activity were associated with lower dysregulation, whereas higher BMI was associated with greater dysregulation. These findings highlight adiposity and physical activity as key modifiable correlates of multisystem physiological burden in older adults.

## Author Contributions

Vishal Vennu conceptualized the study, formal analysis, data curation, visualization, writing–original draft preparation, and writing–review and editing.

## Funding

The work was supported by the Ongoing Research Funding program, King Saud University, Riyadh, Saudi Arabia (ORF‐2026‐1094).

## Disclosure

The funding source had no role in the design, analysis, or interpretation of the study or in the decision to submit the manuscript for publication. The contents are solely the responsibility of the author and do not necessarily represent the official views of the funder.

## Ethics Statement

This study utilized publicly available data from the NHANES 2017–March 2020 cycle. The NHANES protocol received approval from the National Center for Health Statistics (NCHS) Research Ethics Review Board (Protocol #2018‐01) (CDC) https://www.cdc.gov/nchs/nhanes/irba98.htm. Additional ethical approval was not required because this study involved secondary analysis of publicly available de‐identified NHANES data.

## Consent

Each NHANES participant signed a written informed consent form. Additional participant consent was not required because this study involved secondary analysis of publicly available de‐identified NHANES data.

## Conflicts of Interest

The author declares no conflicts of interest.

## Data Availability

The data supporting the findings of this study are publicly available from the National Health and Nutrition Examination Survey (NHANES), National Center for Health Statistics, and Centers for Disease Control and Prevention, at https://www.cdc.gov/nchs/nhanes/?CDC_AAref_Val=https://www.cdc.gov/nchs/nhanes/index.htm.
